# Teaching LGBTQ+ Health, a Web-Based Faculty Development Course: Program Evaluation Study Using the RE-AIM Framework

**DOI:** 10.2196/47777

**Published:** 2023-07-21

**Authors:** Michael Albert Gisondi, Timothy Keyes, Shana Zucker, Deila Bumgardner

**Affiliations:** 1 Department of Emergency Medicine Stanford School of Medicine Palo Alto, CA United States; 2 Stanford School of Medicine Stanford, CA United States; 3 Department of Internal Medicine University of Miami Miller School of Medicine Miami, FL United States; 4 Stanford Educational Technology Stanford School of Medicine Stanford, CA United States

**Keywords:** lesbian, gay, bisexual, transgender, queer, LGBTQ+, queer, faculty development, medical education, continuing education, sexual and gender minority, web-based learning, asynchronous learning, education technology, diversity, equity, inclusion, DEI

## Abstract

**Background:**

Many health professions faculty members lack training on fundamental lesbian, gay, bisexual, transgender, and queer (LGBTQ+) health topics. Faculty development is needed to address knowledge gaps, improve teaching, and prepare students to competently care for the growing LGBTQ+ population.

**Objective:**

We conducted a program evaluation of the massive open online course *Teaching LGBTQ+ Health: A Faculty Development Course for Health Professions Educators* from the Stanford School of Medicine. Our goal was to understand participant demographics, impact, and ongoing maintenance needs to inform decisions about updating the course.

**Methods:**

We evaluated the course for the period from March 27, 2021, to February 24, 2023, guided by the RE-AIM (Reach, Effectiveness, Adoption, Implementation, and Maintenance) framework. We assessed impact using participation numbers, evidence of learning, and likelihood of practice change. Data included participant demographics, performance on a pre- and postcourse quiz, open-text entries throughout the course, continuing medical education (CME) credits awarded, and CME course evaluations. We analyzed demographics using descriptive statistics and pre- and postcourse quiz scores using a paired 2-tailed *t* test. We conducted a qualitative thematic analysis of open-text responses to prompts within the course and CME evaluation questions.

**Results:**

Results were reported using the 5 framework domains. Regarding *Reach*, 1782 learners participated in the course, and 1516 (85.07%) accessed it through a main course website. Of the different types of participants, most were physicians (423/1516, 27.9%) and from outside the sponsoring institution and target audience (1452/1516, 95.78%). Regarding *Effectiveness*, the median change in test scores for the 38.1% (679/1782) of participants who completed both the pre- and postcourse tests was 3 out of 10 points, or a 30% improvement (*P*<.001). Themes identified from CME evaluations included *LGBTQ+ health as a distinct domain*, *inclusivity in practices*, and *teaching LGBTQ+ health strategies*. A minority of participants (237/1782, 13.3%) earned CME credits. Regarding *Adoption*, themes identified among responses to prompts in the course included *LGBTQ+ health concepts* and *instructional strategies*. Most participants strongly agreed with numerous positive statements about the course content, presentation, and likelihood of practice change. Regarding *Implementation*, the course cost US $57,000 to build and was intramurally funded through grants and subsidies. The course faculty spent an estimated 600 hours on the project, and educational technologists spent another 712 hours. Regarding *Maintenance*, much of the course is evergreen, and ongoing oversight and quality assurance require minimal faculty time. New content will likely include modules on transgender health and gender-affirming care.

**Conclusions:**

*Teaching LGBTQ+ Health* improved participants’ knowledge of fundamental queer health topics. Overall participation has been modest to date. Most participants indicated an intention to change clinical or teaching practices. Maintenance costs are minimal. The web-based course will continue to be offered, and new content will likely be added.

## Introduction

### Background

Lesbian, gay, bisexual, transgender, and queer (LGBTQ+) individuals have unique health care needs and face health disparities that are growing in scale [1]. In a 2022 Gallup poll, 7.1% of the US population identified as something other than heterosexual, which is double the percentage of lesbian, gay, bisexual, and transgender (LGBT) respondents to the same poll in 2012 [2]. Now, 1 in 5 Generation Z (born in 1997 to 2012) adults identify as LGBT, which is twice the number of Millennials (born in 1981 to 1996) and far outpaces the reported rates in older generations [2]. If these trends continue, it is likely that >10% of the US population will identify as LGBT within the next several years [2]. However, studies report a physician workforce that is underprepared to care for this growing cohort of Americans [3].

Inadequate physician training in LGBTQ+ health is a remnant of the pathologization of queerness in the 1980s and 1990s during the AIDS crisis [4]. Homophobia and moralistic dialogue in society during that time kept many LGBTQ+ patients from disclosing their sexual orientation to their providers, resulting in substantial unmet care needs [4]. Medical education focused solely on HIV and AIDS at the expense of other LGBTQ+ health topics, such as gender-affirming treatments or medicolegal issues for unmarried couples [5]. This left generations of physicians untrained in queer health, which meant that their students were then similarly untrained [5]. This cyclical failure of medical education has had a ripple effect still felt in our medical schools today [6]. For instance, a study of medical students at 170 US medical schools found that most assessed their training in queer health to be fair or worse [7]. Another study that queried US medical school deans documented a median of 5 hours of LGBTQ+ health content in a 4-year curriculum, with one-third of schools offering no content during the clinical years [8]. Similarly, a survey of US residency program directors in emergency medicine found that only 26% of programs teach LGBTQ+ health; on average, 45 minutes were dedicated to the topic in a 3-year residency program [9]. A study of emergency physicians in Canada found that 97% of participants felt that 2-spirit LGBTQ+ patients deserve the same care as heterosexual patients, and 83% wanted more training [10].

Medical schools must address this training gap. In 2014, the Association of American Medical Colleges published guidelines for teaching LGBTQ+ health in US medical schools and introduced 30 student competencies that would improve the health of LGBT patients [11]. This resulted in new curricula in some schools [12]. However, these training opportunities were generally designed by a small minority of faculty members or students who had expertise or advocacy experience in LGBTQ+ health [13-15]. The average medical school faculty member remains untrained in the basics of queer health, such as accurate vocabulary use (eg, terms related to sex, gender, and sexual orientation), social and behavioral determinants of LGBTQ+ health, medical prevention of HIV, transgender health care, and pelvic health in persons assigned female at birth [5]. Faculty members who lack training in these fundamental domains are underprepared to teach their trainees about the care of LGBTQ+ patients in the clinical setting, perpetuating physician inexperience in these areas. Faculty development is needed for most clinician educators, who are likely underprepared to teach queer health in their daily practice [16]. Descriptions of curricula for faculty training in LGBTQ+ health remain a gap in the medical literature, and this likely reflects the absence of such training in most schools. Only 1 US medical school has published the details of a faculty development program in sexual and gender minority health to date [17].

### Objectives

In 2021, the Stanford School of Medicine released a free, open access, web-based, continuing medical education (CME) course called *Teaching LGBTQ+ Health: A Faculty Development Course for Health Professions Educators* [18] (Multimedia Appendix 1). It is an introductory-level course aimed at clinician educators seeking to improve their knowledge of LGBTQ+ health and the care of LGBTQ+ patients, with a focus on ways to incorporate the course content in their clinical teaching of trainees (Multimedia Appendix 2). It meets the definition of a massive open online course (MOOC) in that the course is available to anyone who wants to take it without charge or limits on participation. In this study, we conducted a program evaluation of the course to understand its impact and inform upcoming revisions and additions to the curriculum.

## Methods

### Study Design

Using a constructivist paradigm, we conducted a program evaluation of the web-based course *Teaching LGBTQ+ Health* using the RE-AIM (Reach, Effectiveness, Adoption, Implementation, and Maintenance) conceptual framework as our guide [19,20]. The purpose of our program evaluation was to understand which types of learners engaged in the course (through participant self-identification as physicians, nurses, other health professionals, or trainees), measure its impact on each group through testing and evaluations, identify any course maintenance concerns, and inform the addition of new course material in upcoming revisions of the curriculum. We defined impact as high user engagement, evidence of learning, and likelihood of practice changes, consistent with elements of the model by Kirkpatrick [21,22]; our specific measures were relevant to web-based learning. We selected the RE-AIM framework as it emphasizes key domains that matched the goals of our evaluation. RE-AIM guides users to evaluate and sustain educational programs such as our course by considering contextual factors to improve public health relevance and population health impact [23]. RE-AIM has been used successfully for the evaluation of MOOCs and other web-based learning courses similar to ours [24,25].

### Study Setting and Population

We conducted this study in 2023 at Stanford School of Medicine (Stanford University, Stanford, California, United States) with data provided by the Stanford Medicine Educational Technology department (Stanford Medicine EdTech), which maintains the course on the internet, and the Stanford Center for Continuing Medical Education (Stanford Medicine CME). Stanford Medicine EdTech instructional designers developed and implemented the course on the internet and were the source of most of the study data. Stanford Medicine CME provided course evaluations from participants who claimed CME credits after course completion. Stanford Medicine CME accredited our course to offer Accreditation Council for Continuing Medical Education, American Nurses Credentialing Center, American Academy of Physician Associates, and American Medical Association continuing education credits. We included data on all participants who completed any section of the course regardless of whether they completed the entire course. We excluded participants who registered for but never started the course.

### Course Design

Our investigator team authored, designed, and built *Teaching LGBTQ+ Health* over a 2-year period from 2019 to 2021. We chose to offer the faculty development course on the web to easily disseminate the content as broadly as possible, and we used closed captioning for all audio elements to increase accessibility. However, we acknowledge that there remain some accessibility challenges inherent to web-based learning platforms. We wrote the course objectives and content based on a needs assessment of novice learners (Multimedia Appendix 3); therefore, the course is limited to introductory material and does not cover advanced topics such as medical and surgical affirming care for transgender patients. We drafted a storyboard that Stanford Medicine EdTech used to build the course platform, illustrate animated characters, develop video content, and create web-based learning activities. We divided the course into four sections: (1) Orientation, (2) Fundamentals of Teaching LGBTQ+ Health (subsections: Introduction, Pretest, LGBTQ+ Health Vocabulary, Social and Behavioral Determinants of LGBTQ+ Health, and Teaching Strategies; Multimedia Appendix 4), (3) Teaching LGBTQ+ Health Cases (including Carla, case of a bisexual woman with a new cancer diagnosis [case 1]; Jesse, case of medical HIV prevention for a serodiscordant couple [case 2]; and Teddy, case of a nonbinary patient seeking affirming pelvic health care [case 3]), and (4) Conclusions, Resources, and CME Credit Instructions (Multimedia Appendix 5). Instructional methods included animated videos, interactive clinical cases, written content, and quizzes. The course was beta tested by an extensive number of Stanford Medicine EdTech staff members and a group of volunteer physicians and medical students outside Stanford; these reviews resulted in the correction of typographical, hyperlink, and caption errors. A second group of experts in LGBTQ+ health also reviewed the course content for accuracy, and no content changes were recommended.

We launched *Teaching LGBTQ+ Health* on the web on March 27, 2021. It is a free, interactive, and self-paced MOOC and requires approximately 90 minutes to complete. Continuing education credits are offered without charge to those who complete the course. *Teaching LGBTQ+ Health* is hosted on the Stanford Medicine Med Education website and supported by Stanford Medicine EdTech. The Med Education learning management system (LMS) is built by Stanford on WordPress (WordPress Foundation) using the LearnDash LMS plug-in (Liquid Web Brand). The interactive components of the course were built using H5P (H5P Group, Flow Coworking), the CM Glossary Tooltip WordPress plug-in (CreativeMinds), and Gravity Forms (Rocketgenius, Inc). Elements of the course can be downloaded by users for free and embedded in other sites via HTML. In February 2023, the course became available on Coursera (Coursera, Inc), a global web-based learning platform that hosts college or university courses, certificates, degree programs, and other MOOCs [26]. We did not edit the content of the course for Coursera; both the Coursera- and Stanford-hosted versions of the course are identical.

### Data Collection and the RE-AIM Framework

We collected data over several weeks from February 2023 to March 2023. We used quantitative and qualitative data analyses to evaluate the course across the 5 RE-AIM domains (Multimedia Appendix 6).

*Reach* refers to participation and demographics. We determined reach based on (1) participation numbers (which included the number of people who registered for the course, participated in any portion of the course, completed the course, or received continuing education credits), (2) demographic data (which included occupation type, area of clinical practice, years in clinical practice, and role or title), and (3) engagement of the target audience (which was defined as Stanford School of Medicine faculty members).

*Effectiveness* is the impact of the course and its effects, both positive and negative. We judged this domain using (1) the results of a 10-question precourse/postcourse quiz (we designed the quiz to align with the learning objectives of each subsection of the course using best practices for item writing (Multimedia Appendix 7) [27], beta tested the quiz among the study authors and later a panel of volunteers who completed the course, and iterated it based on feedback; a standard-setting exercise determined a minimum passing standard of 8 out of 10 correct questions, which were required for successful completion of the course and necessary to obtain continuing education credit [28]; one of the questions purposely did not have a correct answer and was scored as 1 point, so the minimum test score was 1, not zero), (2) a review of CME course evaluations (these included Likert-style responses on a 5-level scale from strongly agree to strongly disagree for statements about the utility of the course content; the effects on the professional growth of the participants; relevance to clinical practice; whether the course had an engaging and interactive format; quality of the content; delivery and effectiveness; value of the topic; overall course rating; and improvement in knowledge, skills, and attitudes; this also included open-text responses to questions about intention to change practice and knowledge and skills learned), (3) sentiment analysis of CME course evaluations and responses to prompts during the 3 clinical cases, and (4) thematic analysis of CME course evaluations.

*Adoption* refers to the ways in which participants can be involved in the intervention. We assessed adoption by performing a (1) thematic analysis of open-text responses to prompts throughout the course (these were questions about how the participant would teach the course content to trainees) and (2) sentiment analysis of clinical case prompts. We also (3) estimated the representativeness of settings and instructors involved in the course.

*Implementation* describes the development and execution of the course. We analyzed this domain through (1) review of costs and grant funding, (2) key project milestones, (3) strategies used for dissemination of course content to other health care organizations, and (4) presentations to potential learner groups internal and external to Stanford.

*Maintenance* refers to the sustainability of the course. For this domain, we evaluated the (1) course platform, (2) continuous quality assurance methods, and (3) plans for the addition of new course content. We also described (4) evidence of institutionalization of the course and related policies in various settings.

### Data Analysis

We analyzed participation rates, quantitative course feedback, and learner demographics using descriptive statistics.

As the pretest and posttest were identical, we performed a paired 2-tailed *t* test to compare learners’ scores (percentage of correct test items) before and after the course. Some participants took the pretest or posttest multiple times, and some of those participants took the posttest repeatedly until they achieved a perfect score. Therefore, we averaged individual participants’ multiple test scores to obtain a single pretest and a single posttest score for statistical analysis. Only participants who completed both the pretest and posttest were included in the analysis. All statistical analyses were performed using the *tidyverse* suite of data analysis tools implemented in the statistical computing language R (version 4.3.0; R Foundation for Statistical Computing) [29,30].

We performed a qualitative thematic analysis of open-text responses and learner feedback following the 6 steps outlined by Braun and Clarke [31]. First, our investigator team met to familiarize ourselves with the type and amount of qualitative data collected. Next, 2 investigators (MAG and SZ) independently coded a portion of the data. They then met to discuss the codes, define them, rename them, and resolve disagreements. This generated the initial codebook. MAG and SZ then coded all the data using the codebook and recorded any new codes that were identified. They reviewed these together once more and made adjustments. Then, the full team met to review the codes and examples from the data to construct potential themes. After discussion, a consensus was reached regarding the final themes, which were named and defined.

In addition, open-text responses and learner feedback were subjected to sentiment analysis using a custom natural language processing pipeline. Specifically, text responses were tokenized into individual words and cross-referenced with the National Research Council Word-Emotion Association Lexicon for emotion and polarity annotation [32,33]. After annotation, the proportion of words associated with each emotion or polarity was calculated for each participant and plotted. Only text responses with ≥5 words were included in the analysis; all others were considered too few for analysis and omitted.

Finally, a program evaluation should not solely report data (ie, *what*) but also offer explanations for the data (ie, *why*). Wherever appropriate, we offer our interpretation of the patterns in the data, potential causes, or implications.

### Reflexivity

We acknowledge that our personal experiences may have biased our analyses. MAG is a senior faculty member, an expert in medical education, and an emergency physician. TK is a MD, PhD student who has extensive experience in LGBTQ+ advocacy and affinity groups as well as deep content knowledge of queer health topics. SZ is an internal medicine resident who has expertise in LGBTQ+ health education and curriculum design. DB is an instructional designer who created the interactive features of the course, oversees its continued maintenance, directs marketing, liaises with continuing education credit providers, and adapts the course to other LMSs such as Coursera. We initially met to discuss our goals for this program evaluation and acknowledged our biases ahead of data analysis. We coded the data based on what was said, not what was inferred. We discussed our biases during coding and theme identification.

### Ethical Considerations

The Stanford School of Medicine Institutional Review Board determined that this study was exempt (IRB-68002). There were no incentives to complete the course and no financial compensation to the course faculty or Stanford Medicine EdTech based on the number of participants in the free course. On the course site, we stated that course data might be used for research purposes. All registration forms contained the Stanford University Privacy Policy [34].

## Results

We report the results of our data analyses using the RE-AIM framework.

### Reach

#### Participation Numbers

As of February 24, 2023, a total of 2577 people had registered for the course, of whom 1782 (69.15%) participants engaged with some of the course content. The 30.85% (795/2577) attrition likely reflects some individuals who registered for the course before continuing education credits were made available (many months after the course launch), decided to wait, and never returned. Only 38.05% (679/1782) of the participants completed the course as defined by the achievement of a minimum passing score on the postcourse exam. The other 61.95% (1103/1782) of participants completed some or all of the course modules but chose not to take the postcourse test, perhaps because they were not eligible for or interested in continuing education credits. Only 13.3% (237/1782) of the participants claimed CME credits; however, CME credits are awarded only to physicians, and this low percentage reflects the many nonphysicians who completed the course as well.

#### Demographics

There were 2 ways to access and register for the web-based course. One method was from the course landing page, which yielded 1544 registrants. Of these 1544 registrants, 1516 (98.19%) provided their *occupation type*—physicians (n=423, 27.9%), students (n=327, 21.57%), and health educators (n=121, 7.98%) represented most participants (Table 1). In addition, 1323 registrants provided their *area of clinical practice*, within which the largest categories were students (n=327, 24.72%), others (n=190, 14.36%), and family medicine and community health (n=139, 10.51%). Participants who self-identified within the “Other” category did not choose an area of clinical practice from the options, perhaps because they were nonstudents and non–health care providers; we did not obtain additional demographic information from this cohort and cannot characterize them further. Of the 423 physicians who reported their area of clinical practice, the largest specialties were emergency medicine and trauma (n=99, 23.4%), family medicine and community health (n=86, 20.3%), and internal medicine (n=60, 14.2%). Finally, 1411 registrants provided their *number of years in clinical practice,* which revealed that most course registrants were either still in training (n=578, 40.96%) or within <5 years of having finished training (n=254, 18%; Table 2).

The other method available for course registration was through the Stanford Medicine EdTech Med Education LMS, which yielded 1333 registrants and different demographic questions. Most of these participants were physicians (473/1333, 35.48%) or students (364/1333, 27.31%). Of these participants, 46.96% (626/1333) identified as “in training” when asked about their years in clinical practice; this cohort likely included resident physicians, fellows, and other “nonstudent” trainees. Another 21.16% (282/1333) of participants identified as being within 5 years of completion of their training.

**Table 1:** Participants’ reported professions (n=1386).

Profession	Participants, n (%)
Physician	423 (30.52)
Student	327 (23.59)
Health educator	121 (8.73)
Other	115 (8.3)
Psychologist	71 (5.12)
Nurse	55 (3.97)
Researcher	48 (3.46)
Non–health care provider	47 (3.39)
Social worker	40 (2.89)
Nurse practitioner	34 (2.45)
Health care administrator	30 (2.16)
Physician assistant	25 (1.8)
Other hospital staff	22 (1.59)
Pharmacist	10 (0.72)

**Table 2:** Participants’ experience in years of practice (n=1411).

Years of practice	Participants, n (%)
In training	578 (40.96)
<5	254 (18)
5-10	206 (14.6)
11-20	202 (14.32)
21-30	103 (7.3)
≥31	68 (4.82)

#### Engagement of the Target Audience

This faculty development course was designed for educators across the health professions, although Stanford faculty members were the target learners. A minority of participants (64/1516, 4.22%) had a Stanford University email address. Gmail addresses were the most commonly used, and it is unknown how many Stanford affiliates used their personal email addresses rather than their Stanford email addresses. Therefore, the number of Stanford participants was likely higher. Of the 2610 physician faculty members of the Stanford School of Medicine, no fewer than 18 (0.69%) completed the course. Similarly, this number resulted from a review of email addresses and represents a minimum. We assumed that more Stanford affiliates would complete the course simply because it was created at our institution. Reasons for low engagement potentially included the lack of incentives, perception that the course material was not relevant, saturation of professional development opportunities offered at our institution, or poor marketing. We did not set a target participation rate, although we expected more learners from Stanford to participate.

### Effectiveness

#### Precourse and Postcourse Quiz

A total of 65.43% (1166/1782) of the participants completed the precourse quiz, and 35.63% (635/1782) attempted the postcourse quiz. We suspect that this attrition is related to the desire to obtain continuing education credits as the posttest was a requirement and not all users pursued credits. Alternatively, this attrition may represent users who simply did not complete the course for any reason, such as the lack of interest or time.

**Figure 1:**
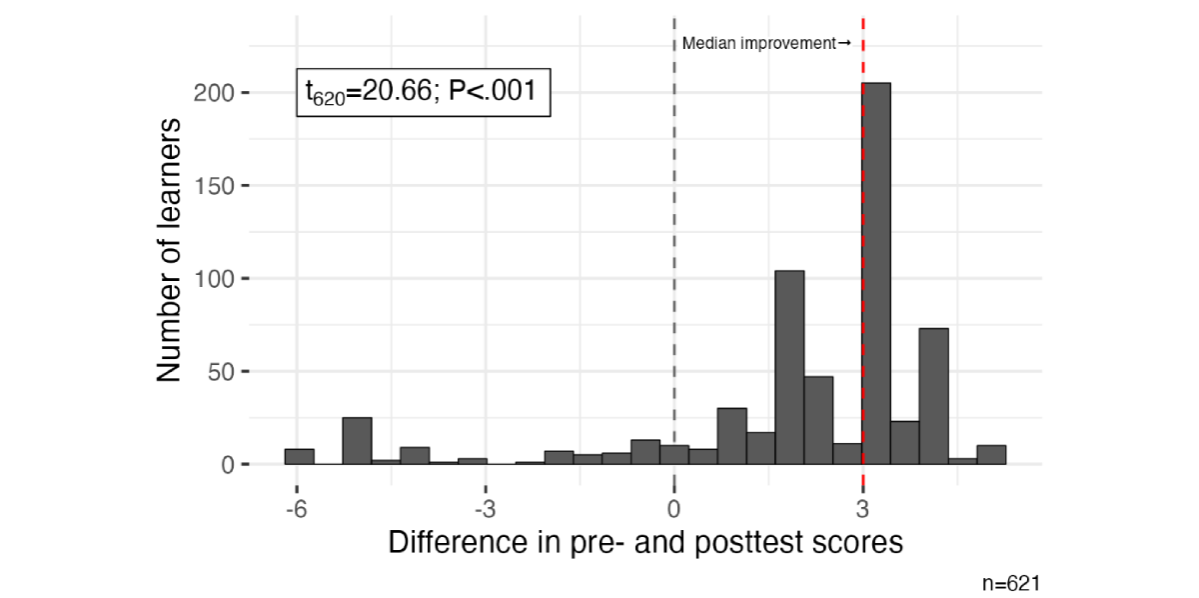
Histogram of the difference in pre- and posttest scores (posttest score – pretest score) for course registrants. The gray dashed line indicates the null hypothesis (that the average change in scores is 0), and the red dashed line indicates the median observed change in scores. Most of the observed distribution of score differences lies to the right of 0—this indicates that most participants’ scores were higher on the posttest than on the pretest.

None of the participants passed the pretest on their first attempt, and 67% (431/643) of the participants met the minimum passing standard on the posttest on their first attempt. The pretest scores are notable as we designed *Teaching LGBTQ+ Health* as an introductory-level course; all participants failed the pretest regardless of background and years of training and practice. The median number of postcourse quiz attempts was 1. The median change in test scores was 3 out of 10 points, or a 30% improvement (t_678_=20.44; *P*<.001; Figure 1). Most participants across all major subgroups improved their scores similarly; for instance, students and practicing physicians had nearly the same median change in scores (2.5 for students and 3.0 for physicians). This analysis indicates that the course effectively improved participant knowledge related to our course objectives across all learner subgroups as the course objectives mapped directly to the 9 scored questions on the test.

#### CME Course Evaluations

**Figure 2:**
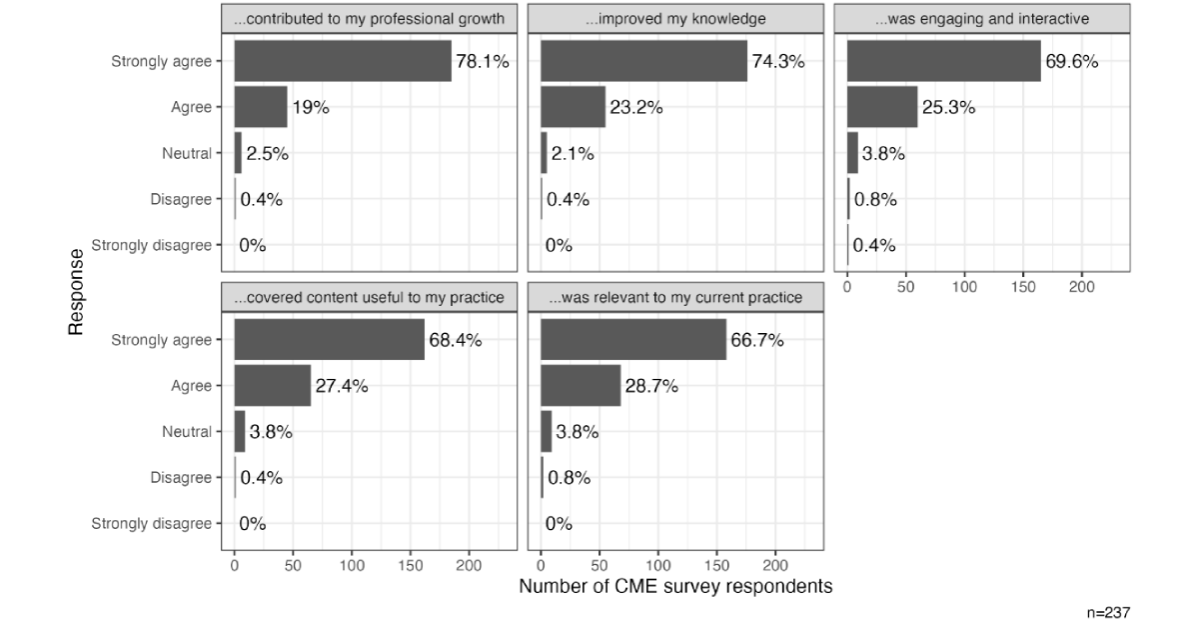
Continuing medical education (CME) evaluations: overall ratings; responses to the prompt, How much do you agree with the following statement, “This CME activity...?”.

**Figure 3:**
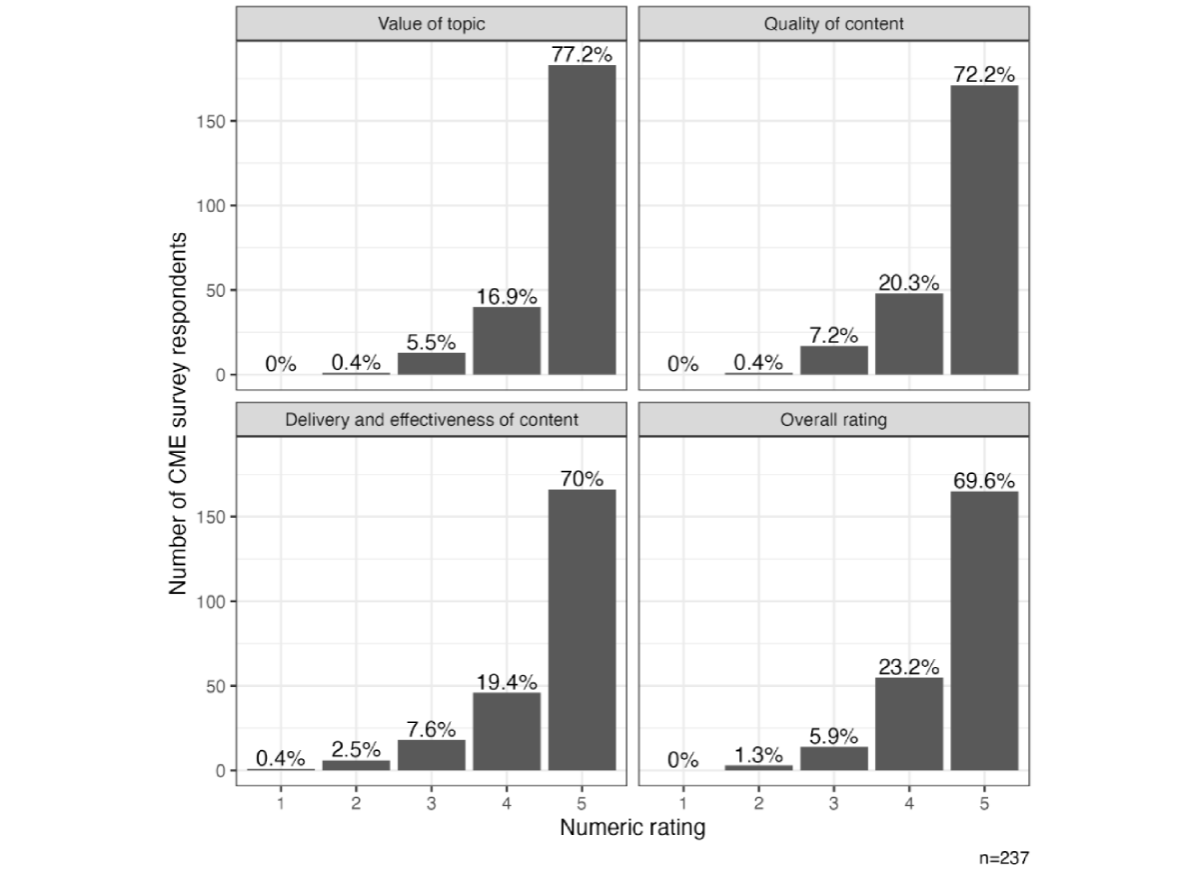
Continuing medical education (CME) evaluations; responses to the prompt, Rate each component of the course on a 1-5 scale.

We analyzed CME course evaluations from the 13.3% (237/1782) of physician participants who completed the course and claimed CME credit. Most participants strongly agreed with positive statements about the course design and effectiveness (Figures 2 and 3). Overall, the course evaluations were outstanding for each of the survey items, as illustrated in the figures.

#### Sentiment Analysis

**Figure 4:**
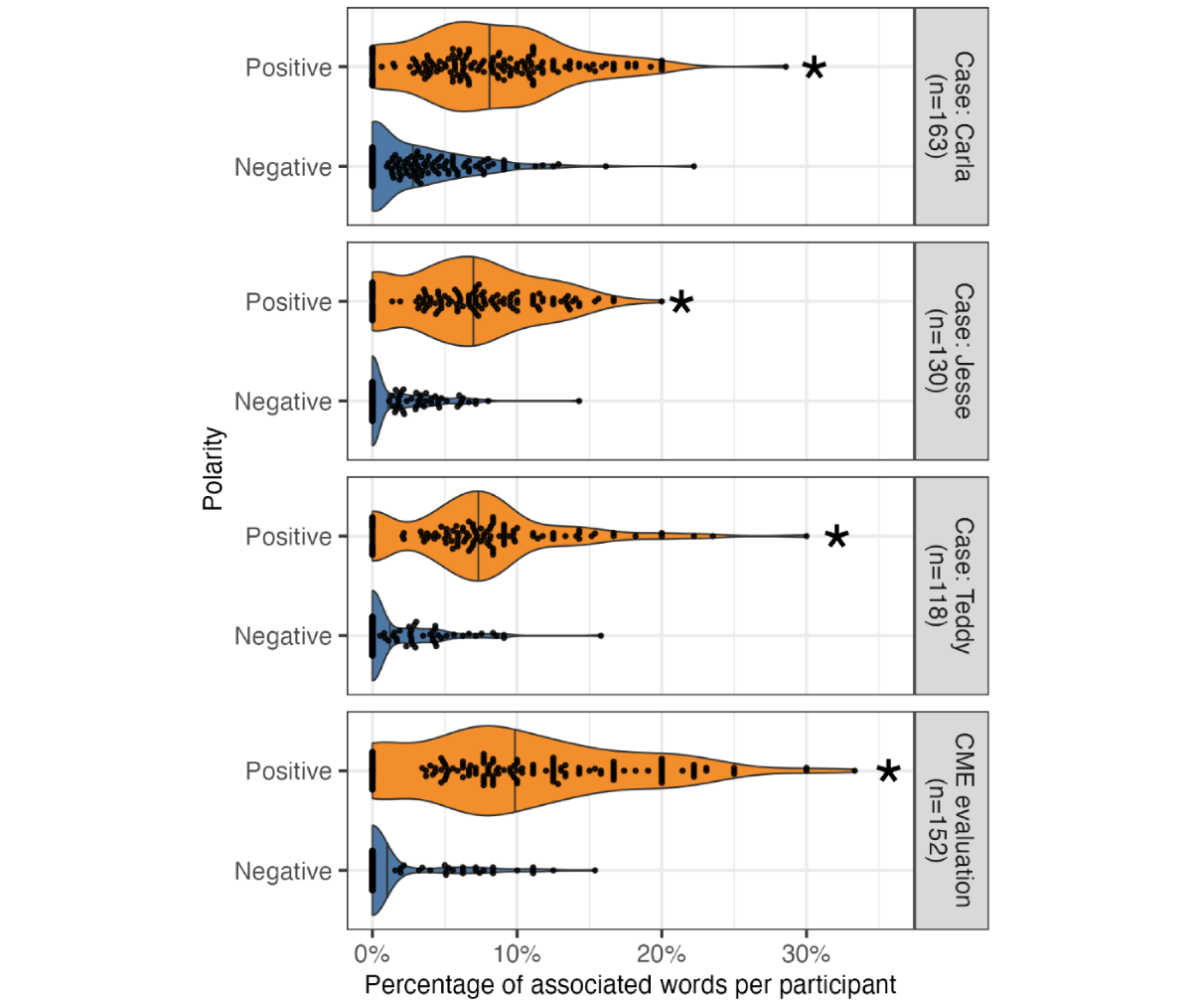
Sentiment analysis of free-text responses revealing positive polarity. Violin and beeswarm plots illustrating the proportion of words associated with positive and negative polarity in each participant’s free-text responses in the continuing medical education (CME) evaluation (top), Carla patient case (second), Teddy patient case (third), and Jesse patient case (bottom). Each dot represents a single participant, vertical lines represent the median proportion for a given distribution, and asterisks indicate statistical significance at the level of .05 using the Wilcoxon rank sum (Mann-Whitney) test.

The strength of the course evaluations was further supported by quantitative sentiment analysis of open-text responses on both the CME evaluation and in response to the course’s case presentations (Figure 4). Overall, positive sentiments were significantly more prominent than negative sentiments, with statistically significant differences in the CME evaluation (Mann-Whitney *U*=215; n_1_=n_2_=152; *P*<.001), the “Carla” case presentation (Mann-Whitney *U*=1225; *P*<.001), the “Teddy” case presentation (Mann-Whitney *U*=129; n_1_=n_2_=118; *P*<.001), and the “Jesse” case presentation (Mann-Whitney *U*=212; n_1_=n_2_=130; *P*<.001) [34].

#### Thematic Analysis of CME Course Evaluations

We identified 3 themes from the open-text responses to questions on the CME evaluation (Table 3). The questions asked about anticipated changes in practice and new knowledge, skills, or attitudes acquired from the course. These themes included (1) “LGBTQ+ Health as a Distinct Domain” (the acknowledgment by participants that LGBTQ+ health is a unique body of knowledge and skills), (2) “Inclusivity in Practices” (the use of communication techniques [clinical and teaching] and clinic design to ensure LGBTQ+ patients and students feel welcome and respected), and (3) “Teaching LGBTQ+ Health Strategies” (the variety of instructional techniques that can be used to teach this material). These data demonstrate the effectiveness of the course in changing provider perspectives, teaching content, and refining skills.

**Table 3:** Thematic analysis of continuing medical education course evaluations.

Theme	Operational definition	Representative quotes
LGBTQ+^a^ health as a distinct domain	LGBTQ+ health is a distinct body of knowledge and related skills that requires intentional training and provider competence.	“I plan to incorporate more inclusive language; I understand what the LGBTQ+ community really means and inviting them to receive quality primary health care with warmth so that feel comfortable.” “I intend on using gender affirming language in all of my clinical encounters and educate others on LGBTQ+ health so that there is more awareness and understanding of the disparity of health outcomes for LGBTQ+ patients.”
Inclusivity in practices	Routine clinical, nonclinical, and teaching practices must be inclusive of sexual and gender minority groups. Examples include the correct use of vocabulary, the design of inclusive clinical environments, and the practice of affirming care in clinical encounters.	“Incorporating appropriate language and use of pronouns in case studies, exams, and role play activities. Being more aware of gender issues during teaching, modeling behaviors and language for students.” “I will make adjustments to our student and faculty Allies trainings and, when training others, incorporate this material, including terminology and case scenarios, to more fully equip students and faculty at my institution with the skills and competencies necessary to treat LGBTQ+ patients more equitably.”
Teaching LGBTQ+ health strategies	LGBTQ+ health content can be included in most routine teaching activities throughout health profession schools. Modalities include the addition of new LGBTQ+ health content to courses, role modeling of the 5 P’s of sexual health and correct vocabulary use, and new item writing for tests.	“Small group discussions about the difference between health seeking in case of familial rejection for LGBTQ+ individuals as compared to the majority community can be highlighted.” “I would emphasize the 5Ps of sexual health and have the students role play about taking sexual history, identify difficult questions and guide them on how to handle these circumstances.”

^a^LGBTQ+: lesbian, gay, bisexual, transgender, and queer.

### Adoption

#### Thematic Analysis of Open-Text Responses

We analyzed the responses to prompts in the course that queried how participants would teach the course content to their trainees (Table 4). Themes identified were (1) “LGBTQ+ Health Concepts” (participants reported key concepts of queer health that they learned in the course and that would inform their teaching practices) and (2) “Instructional Techniques” (participants identified 2 instructional methods that must be used to teach LGBTQ+ health). These themes broadly reflected changes in participants’ knowledge, skills, and attitudes that resulted from the course. We identified subthemes that represented “LGBTQ+ Health Concepts” that participants planned to teach their students using specific “Instructional Techniques.” The subthemes are listed in Table 4 with definitions and representative quotes. These data indicated ways in which participants planned to incorporate what they learned into their clinical and teaching practices, an important measure of adoption.

**Table 4:** Thematic analysis of open-text responses to the following prompt: “How would you teach this course content?”

Theme and subtheme		Operational definition	Representative quotes
**LGBTQ+^a^ health concepts: unique health needs of queer and gender-diverse patients**			
	Social and behavioral determinants of health	Environmental, situational, and behavioral characteristics affect health care access and a wide range of health outcomes. With respect to this course and the care of queer patients, examples include substance use, minority stress, familial rejection, access to health care, sexual practices, and victimization.	“[I will] be more mindful in incorporating social and behavioral determinants of LGBTQ health, and how it relates to negative health outcomes and sequelae.” “[I will] incorporate social determinants of health in LGBTQ+ teaching with all levels of learners.”
	Medicolegal issues	Unique legal challenges and health disparities experienced by sexual and gender minority groups, whether individually or as couples. Examples include advance directives, surrogate decision maker designations, and visitor policies.	“When we discuss advance directives, we can specifically incorporate LGBTQ+ patients.” “[I will] teach students to ask about trauma (both physical and mental) and to review chart and make sure there is advanced directives, teaching students about legal issues that may affect LBGQT+ populations, in particular.”
	Chosen families	Chosen families consist of nonbiological individuals who have deep bonds of support and mutual love. As reflected in our Teaching LGBTQ+ Health cases, chosen families result from engagement in supportive communities or rejection by nuclear families.	“Teaching about a person’s chosen family and prioritization of healthcare proxies that may not be legally recognized in certain states is an important thing to learn as a physician.” “[I will teach] clinical simulations where students can gain experience having discussions around advanced directives, priority lists, and chosen family.”
	Sexual health	LGBTQ+ health is more than just HIV and AIDS. It includes a global discussion of sexual practices and behaviors that affect health, among other topics. An example from our course includes the use of the CDC^b^ 5 P’s of sexual behavior history taking.	“The 5 P’s of sexual health should be included in history taking from the beginning of medical school. A lot of times the sexual history is brushed over, but it is important to obtain this information from the onset.” “I will make a presentation about my students with the 5 Ps model and explain to them why we need to use this technique when speaking with all patients about their sexual health. I will then select a few patients and test my students with those patients.”
	Affirming care	Clinical practices that respect sexual and gender minority groups. Examples include the use of nonjudgmental and inclusive interviewing techniques, correct vocabulary, inclusive clinical environments, and trauma-informed care.	“[I will] Incorporate elements of safe space in office, such as Queer Patient Bill of Rights, educating front desk staff on gender affirming language, having signs that suggest LGBTQ plus welcome, and installing gender neutral bathrooms.” “When teaching the pelvic exam to students, it would be important to teach the effects of gender-affirming medical therapies (such as testosterone) on the exam and how to provide trauma-informed care.”
**Instructional techniques: the maturation of daily, routine teaching activities to include LGBTQ+ health**			
	Role modeling	A teaching modality that is historically important in medicine and timeless and requires faculty competence. Key is the demonstration of “how to say” and “how to do” simultaneously to students, generally at the bedside.	“The suggestion for role modeling is absolutely a good first step. These are conversations that students would typically approach with caution, so it would be important to show them how to have these conversations.” “Modeling how to take a sexual history can be very helpful for students and learners. This can be accomplished by discussing the ‘5 Ps’ methodology and practicing this method when obtaining sexual histories.”
	Student practice	Students need distinct opportunities to practice what they learn in safe spaces, with feedback from trained faculty, and before clinical encounters.	“I would follow the ‘see one, do one, teach one model,’ first modeling the use of the 5 P’s, then observing my student using them and provide feedback, and then once they feel comfortable, encourage them to teach the skill to others.” “During my school’s introduction to clinical medicine course, we practiced the 5 P’s of Sexual Health and were given the opportunity to practice our communication with transgender patients. I thought that this was a really great opportunity to learn about how to make patients feel comfortable when asking questions about sexual history.”

^a^LGBTQ+: lesbian, gay, bisexual, transgender, and queer.

^b^CDC: Centers for Disease Control and Prevention.

#### Sentiment Analysis of Clinical Case Prompts

**Figure 5:**
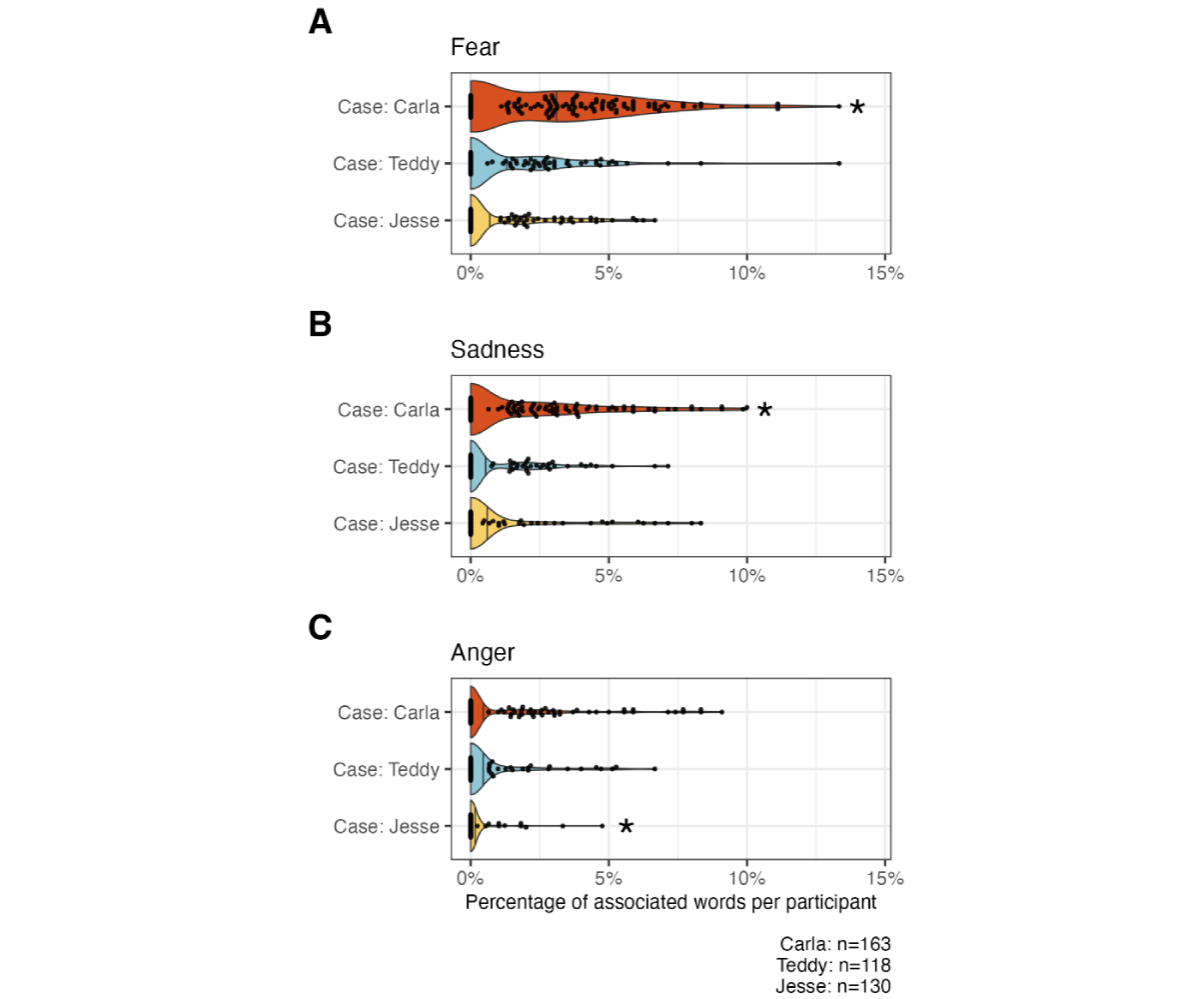
Sentiment analysis revealing that participants’ free-text responses successfully reflected the course’s case-based learning goals. Violin and beeswarm plots illustrate the proportion of words associated with (A) fear, (B) sadness, and (C) anger in each participant’s free-text responses in case report evaluations. In all panels, each dot represents a single participant, and vertical lines represent the median proportion for a given distribution. The asterisks indicate statistically significant enrichment in fear-, sadness-, and anger-associated words, respectively relative to the other cases at the level of .05 using the Wilcoxon rank sum (Mann-Whitney) test.

Further evidence that participants internalized these core themes during the course can be seen in the sentiment analysis of their free-text responses to each of the 3 cases (Figure 5). In particular, the “Carla” case presentation (a bisexual woman with a new cancer diagnosis) emphasized many of the more challenging themes of the course, including familial rejection, trauma, and mortality associated with poor access to care (Multimedia Appendix 8). Accordingly, sentiment analysis revealed a statistically significant increase in *fear*- and *sadness*-associated words—such as “rejection,” “abuse,” and “trauma”—in the free-text responses to “Carla” compared with the responses to “Jesse” (Mann-Whitney *U*=14,438; n_Carla_=163; n_Jesse_=130; *P*<.001) and “Teddy” (Mann-Whitney *U*=12,320; n_Carla_=163; n_Teddy_=118; *P*<.001). In contrast, no difference was observed between “Jesse” and “Teddy” (Mann-Whitney *U*=8431; n_Teddy_=118; n_Jesse_=130; *P*<.001). Similarly, the “Carla” and “Teddy” (a nonbinary patient seeking affirming pelvic care) case presentations incorporated themes involving discrimination and unfair treatment in the clinical environment, whereas the “Jesse” case (a gay male patient considering medical HIV prevention strategies) centered on a patient with largely positive experiences with his primary care provider (Multimedia Appendix 9). This difference in context between the cases was reflected in participants’ free-text responses to the “Jesse” case, which contained significantly fewer *anger*-associated words—such as “discrimination” and “bias”—than both the “Carla” (Mann-Whitney *U*=12,766; n_Carla_=163; n_Jesse_=130; *P*<.001) and “Teddy” (Mann-Whitney *U*=8688; n_Teddy_=118; n_Jesse_=130; *P*<.001) cases. Together, these data suggest that the participants learned the key features of each case presentation.

#### Representativeness of Settings and Instructors

We did not ask participants about their sexual orientation or gender. Demographic data suggest that the course appealed to both student and faculty audiences despite being marketed as a faculty development program. Future iterations of the course will be designed to appeal more broadly to nonphysician audiences. We partnered with the Medical Student Pride Alliance to recruit volunteers to provide character voices in course videos, beta test the course before launch, and promote the course on the web [35]. Our guiding principle during course development reflects representativeness: “nothing about us, without us” [36]. The course is well regarded among the LGBT training opportunities at Stanford Medicine.

### Implementation

#### Costs and Grant Funding

The cost of developing the course was divided into direct charges to support the efforts of Stanford Medicine EdTech designers and programmers and uncompensated efforts by course faculty, volunteers, and administrative staff. The direct charges totaled approximately US $57,000. This was funded by an education innovations research grant from the Stanford Medicine Teaching and Mentoring Academy (US $19,701), a subsidy from Stanford Medicine EdTech (US $15,000), and the Precision Education and Assessment Research Lab in the Stanford Department of Emergency Medicine (US $22,299). The Department of Emergency Medicine provided administrative support. Course faculty (1 medical school faculty member and 2 medical students) collectively spent >600 hours preparing the course. Additional volunteers included voice actors (5 total, 20 total hours worked), expert review of the course (2 work hours), and beta testing by physician volunteers (6 work hours). On the basis of these experiences, the projected direct cost of adding a new 10-minute animated case module to the existing course platform is US $17,000 in 2023.

#### Key Project Milestones

Over 2 years, we have achieved the following key milestones: grant submission and funding, securing Stanford Medicine EdTech collaboration, needs assessment, delineation of learning goals and course objectives, content and script finalization, storyboarding, character animation, custom visual development, audiovisual editing, beta testing, launch communications and webinars, marketing, continuing education accreditation, distribution to Coursera, and program evaluation. A similar cycle of key milestones can be expected for any additional course content to be developed.

#### Strategies for Dissemination of the Course

We used social media, CME listserves, and cross-marketing with another Stanford web-based course to publicize our course (Multimedia Appendix 10). We contacted LGBT health organizations in major US cities and large cities in English-speaking countries, notified LGBT news organizations in the United States, and did direct outreach to medical professional societies. We used a snowball technique in which we asked participants to share the course with their colleagues and someone outside their institution, and we made the course searchable on the internet. Now, the course has also been made available on Coursera, which has substantially increased participation in the several weeks between Coursera launch and the preparation of this manuscript (several hundred new participants in <2 months; not analyzed in this study).

#### Presentations to Potential Learners

We presented the course to live audiences via Zoom (Zoom Video Communications, Inc) for educational and marketing purposes. The course is animated and interactive and, therefore, lends itself well to live demonstrations of functionality, content, and user experience. It is more visually appealing than many other web-based CME courses, which we hoped would dispel biases about web-based learning. Some of the initial audiences included the National LGBTQ Health Awareness Week; a women’s organization within the US Navy; the Stanford Ethics, Society, and Technology Hub Unconference; a case study presentation on how to build robust web-based courses for the 2021 Stanford Medicine CME Live Conference; a webinar for educators from historically Black colleges and universities in the United States; numerous medical school grand rounds lectures; and internal Stanford Medicine department presentations.

### Maintenance

#### Course Platform

The course continues to be hosted on the Stanford Med Education LMS at a cost of US $1000 per year, subsidized by the Stanford Medicine EdTech department. We have not had to make any adjustments to the platform since the course launch 2 years ago. Regular maintenance and troubleshooting support are supplied as needed by the Stanford Medicine EdTech department.

#### Continuous Quality Improvement

We closely monitor course feedback to ensure that the LMS is functioning properly and identify any content that needs to be edited. No signals have resulted in a change to the course yet, although a medication recommendation will be modified this year. Course evaluations have remained very positive throughout the 2 years that the course has been on the web, and we believe that the course content remains up-to-date and relevant.

#### Plans for Additional Content

The course was launched in March 2021, and if successful, the goal was to add new content by 2024. We conducted this program evaluation to determine whether that plan should continue. We have met with numerous stakeholders within the Stanford LGBTQ+ enterprise as a needs assessment for new content within our institution. There is a demand for additional course modules regarding the care of transgender patients, especially adolescents.

#### Institutionalization

A large number of trainees completed this faculty development course. We know of 1 US medical school that requires preclinical students to complete the course, likely explaining this observation. We also noted a very large number of registrants with the same email address from another US medical school, which suggests that the course was likely required for this cohort of participants as well. We are unaware of other mandated audiences or policies related to this course. The Stanford offices that funded the course continue to market it regularly. We anticipate that this study will facilitate future institutionalization and incentivization for completion at our medical school.

## Discussion

### Principal Findings

We conducted a rigorous program evaluation of the *Teaching LGBTQ+ Health* course that provided an understanding of its impact to date and informed our decisions about the course moving forward. We assessed impact using many measures of course engagement, evidence of learning, and likelihood of practice change [21,22]. Although we found a low participation rate by the target population, we were pleased with the degree of course engagement outside our institution and across disciplines, with excellent participant feedback. Participation beyond the Stanford School of Medicine spoke to our a priori decisions to make the course free, open access, and available for continuing education credits for physicians and nurses. The analysis of our pre- and postcourse quiz and CME course evaluations provided evidence of effective learning. Our thematic analysis identified meaningful ways in which participants intended to change their clinical or teaching practices based on the course content; we hope that such actions ultimately translate into improved care for LGBTQ+ patients. Sentiment analysis confirmed that most participants achieved the learning goals of the interactive clinical cases. Therefore, our summary appraisal is that the course has been impactful, recognizing that action is needed to increase reach. With proper marketing and incentivization of faculty participants, we believe that the course can be successfully implemented to scale at many different health profession schools.

We believe that *Teaching LGBTQ+ Health* is a unique learning resource for health profession educators that fills an important training gap [16]. It was purposely designed as a faculty development course that would simultaneously provide an introduction to LGBTQ+ health content and methods of teaching that content to trainees. Faculty development programs aimed at improving the teaching of queer health content are rarely described and are primarily found in the nursing literature [37,38]. However, Harvard Medical School recently published a comprehensive sexual and gender minority health curriculum for medical students that included an impressive faculty development plan; notably, they used web-based learning modules somewhat similar to our course [17]. Most other published LGBTQ+ health curricula or curriculum mapping exercises have been used in undergraduate or graduate medical education programs but not for CME or faculty initiatives [3,12,39,40]. We believe that faculty training—not just student training—is critical for the normalization of LGBTQ+ health content in the routine teaching activities of our schools. The Stanford and Harvard web-based courses can provide free faculty development on queer health to other health professionals, most of whom practice at medical centers that do not offer such faculty training.

We separately analyzed 2 collections of qualitative data, one sourced from CME course evaluations and the other from open-text responses to prompts throughout the course. These data reflected learned content (*Effectiveness* domain in RE-AIM) and intentions to change (*Adoption* domain), and the themes that we identified aligned well with these concepts. Interestingly, the themes discerned from each data set were quite similar, representing 3 broad findings. First, participants recognized LGBTQ+ health as a distinct body of knowledge, a notion that is well established in the literature but may have been new to novice learners in our course [41-43]. Of note, only practicing physicians and not trainees complete CME evaluations, so it can be surmised that faculty participants were those who were the most struck by the scope of LGBTQ+ health content. Second, of the instructional methods reviewed in the course, role modeling stood out as particularly important; this is consistent with the historical use of role modeling as a classic bedside teaching technique [44,45]. Faculty would be wise to incorporate role modeling when teaching new trainees, especially with respect to initially challenging communication skills such as obtaining a comprehensive sexual history or counseling patients about end-of-life decisions. Finally, participants intended to be more affirming and inclusive in their future practices, which represented an important change in attitudes and skills that resulted from the course. Affirming care practices are critically important for the treatment of sexual and gender minority groups’ health concerns and have been shown to improve patient care [46,47].

MOOCs are often evaluated using measures of learning, learner engagement, and learners’ experiences interacting with the platform [48]. We examined these measures in a variety of ways using the RE-AIM framework to guide our program evaluation. The RE-AIM domains included these important measures and many others and aligned well with our stated goals for the exercise. Similar to other studies of MOOCs, the use of both quantitative and qualitative data in a RE-AIM evaluation resulted in a robust set of inputs and outputs to examine [24]. These data are voluminous, which therefore requires this lengthy summary report. We found that RE-AIM was a valuable method to gather the broad data we needed for informed decisions regarding our course. We believe it to be a practical and effective framework that can be useful when conducting a program evaluation of a curriculum of any size.

Program evaluation outcomes may be instrumental (used to make improvements or changes), conceptual (used to evolve an understanding of a program but without changing it), or symbolic (used when an evaluation is required for justification of a change or for reporting purposes) [49]. We conducted this evaluation for instrumental purposes, specifically to answer whether the course should continue to be offered and should be expanded. We confirmed that the course is inexpensive to maintain on the internet; therefore, we will continue to offer it. However, it was very costly to develop. New modules designed to match the current course esthetic will again require significant funding. However, as the course LMS site has already been built, it is much easier and less expensive to develop new content. We will apply for new grant funding for this purpose. Key design and implementation milestones for new content development will mirror those of the initial course, as described previously in the *Key Project Milestones* subsection of the *Results* section. Other health profession educators interested in developing similarly interactive and animated web-based courses should be aware of the costs involved.

### Limitations

There were several important study limitations. Registration and participation numbers may be misinterpreted as the primary measures of a successful course (ie, an assumption that more participation means more impact). Although reach is very important for the program evaluation of an MOOC, it does not assess course quality and, therefore, would limit our understanding of impact if considered alone. The additional domains of RE-AIM offered a richer understanding of impact in this study. However, we acknowledge that course participation may be overemphasized by our stakeholders and readers. Only a subset of the participants completed the pre- and postcourse quiz or provided course feedback; we must assume that participants who did not fully engage in the course were less affected by it. The length of the quiz—only 10 questions—may not have been discriminating enough to fully appreciate the degree of participant learning, although our data were statistically significant (t_678_=20.44; *P*<.001) and implied its effectiveness. In addition, we do not have longitudinal data from participants about changes in their practice habits; we only have intention-to-change data. Finally, the evaluation of MOOCs is subject to biases that result from potentially large and diverse groups of learners; these biases are somewhat mitigated by the use of pre- and posttests, as in our study [48]. However, we did not control for other confounding variables related to these biases.

In summary, our evaluation of *Teaching LGBTQ+ Health* suggests that it was an expensive and time-consuming course to create, was impactful, met its learning objectives for those who completed the course, missed its target audience but had broad appeal, and requires very little ongoing maintenance. On the basis of this evaluation, the course will continue to be offered by Stanford Medicine EdTech and Coursera, and we plan to include additional content if appropriate funding is identified. Our goal is to use the web-based platform as a flagship for a suite of LGBTQ+ health curricula; this program evaluation was viewed as foundational to such an initiative.

### Conclusions

*Teaching LGBTQ+ Health* improved participants’ knowledge of fundamental queer health topics. Overall participation has been modest to date. Most participants indicated an intention to change their clinical or teaching practices. Maintenance costs are minimal, and the course will continue to be offered on the web for free. New content is likely to be added.

## Appendix

Multimedia Appendix 1 *Teaching LGBTQ+ Health* course logo. LGBTQ+: lesbian, gay, bisexual, transgender, and queer.

Multimedia Appendix 2 *Teaching LGBTQ+ Health* course trailer. LGBTQ+: lesbian, gay, bisexual, transgender, and queer.

Multimedia Appendix 3 Learning objectives.

Multimedia Appendix 4 Didactic clip: sexually transmitted infections.

Multimedia Appendix 5 Curriculum summary.

Multimedia Appendix 6 Data sources for the Reach, Effectiveness, Adoption, Implementation, and Maintenance evaluation of the *Teaching LGBTQ+ Health* course. LGBTQ+: lesbian, gay, bisexual, transgender, and queer.

Multimedia Appendix 7 Pre- and postcourse test questions and answers.

Multimedia Appendix 8 Carla case clip.

Multimedia Appendix 9 Didactic clip: pre-exposure prophylaxis.

Multimedia Appendix 10 *Teaching LGBTQ+ Health* press release. LGBTQ+: lesbian, gay, bisexual, transgender, and queer.

## Abbreviations

CME: continuing medical education
LGBT: lesbian, gay, bisexual, and transgender
LGBTQ+: lesbian, gay, bisexual, transgender, and queer
LMS: learning management system
MOOC: massive open online course
RE-AIM: Reach, Effectiveness, Adoption, Implementation, and Maintenance
Stanford Medicine CME: Stanford Center for Continuing Medical Education
Stanford Medicine EdTech: Stanford Medicine Educational Technology department

## Data Availability

The codebook for the demographic and quantitative analysis is located on the following GitHub repository [50]. The qualitative data sets analyzed in this study are available from the corresponding author upon reasonable request.
