# Supplementary figures and images for: Teaching LGBTQ+ Health, a Web-Based Faculty Development Course: Program Evaluation Study Using the RE-AIM Framework
Source: JMIR Med Educ. 2023 Jul 21;9:e47777. doi: 10.2196/47777 (PMC10403800; doi:10.2196/47777)

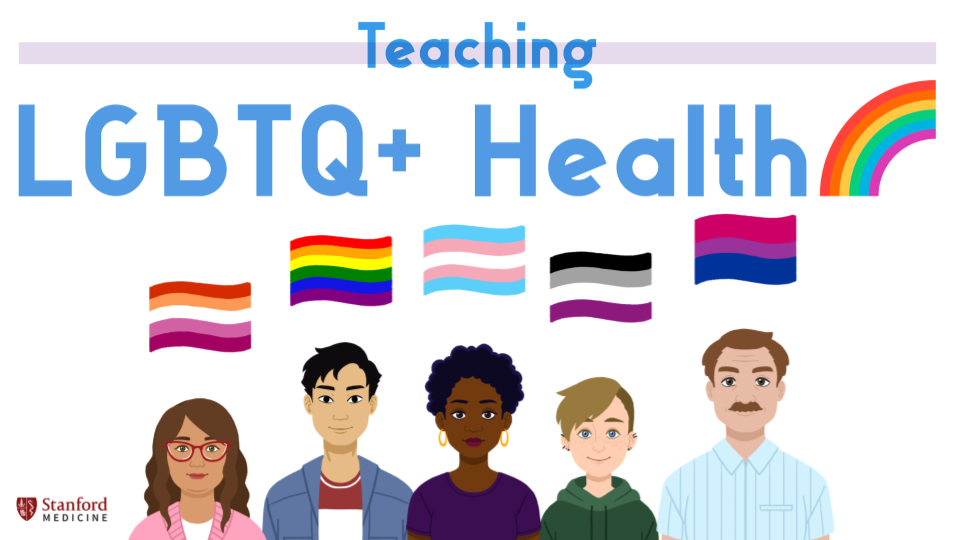

Supplement: Multimedia Appendix 1 [file mededu_v9i1e47777_app1.png]

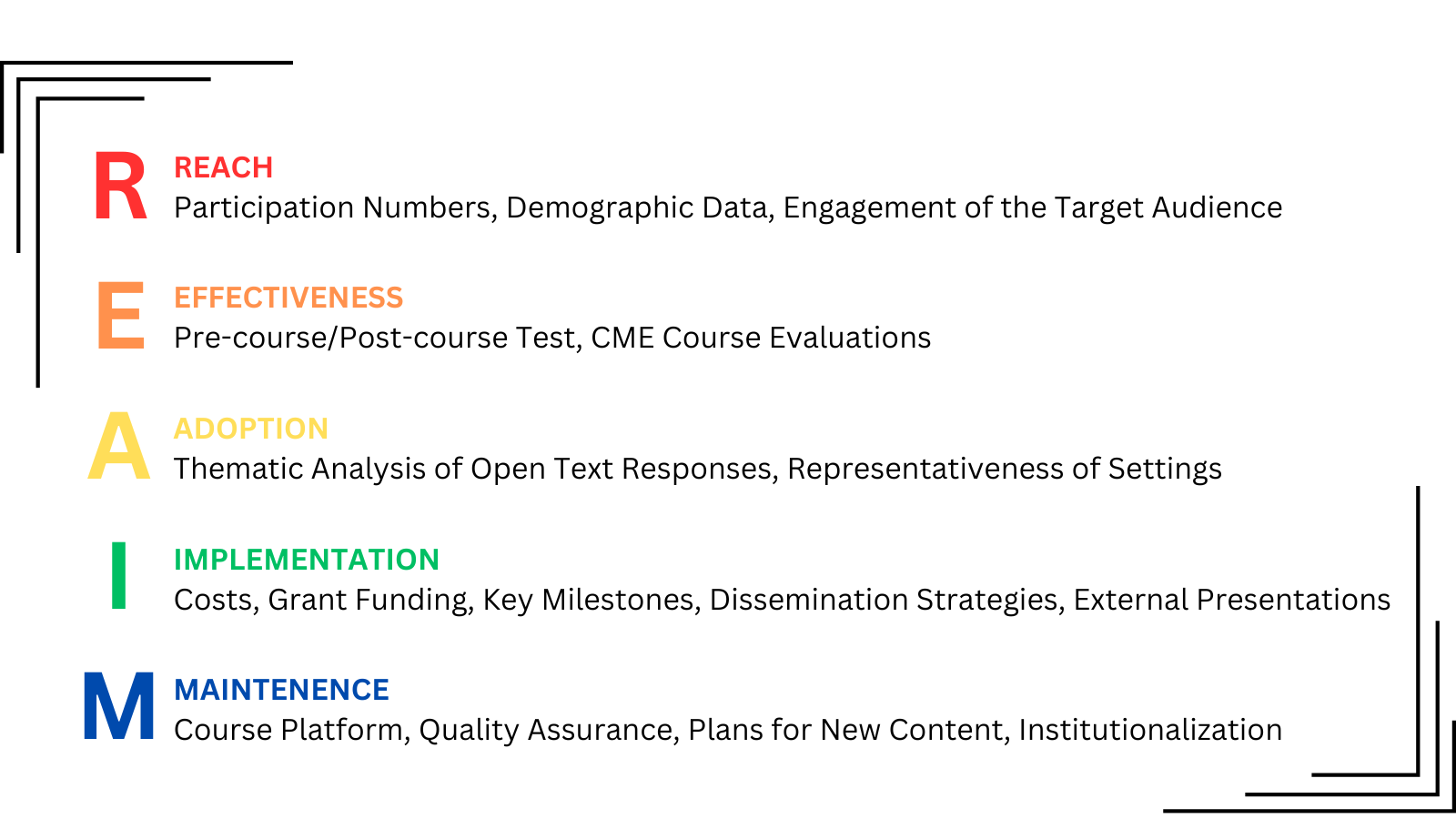

Supplement: Multimedia Appendix 6 [file mededu_v9i1e47777_app6.png]
