# Supplementary material for: Teaching LGBTQ+ Health, a Web-Based Faculty Development Course: Program Evaluation Study Using the RE-AIM Framework
Source: JMIR Med Educ. 2023 Jul 21;9:e47777. doi: 10.2196/47777 (PMC10403800; doi:10.2196/47777)
Supplement: Multimedia Appendix 3 [file mededu_v9i1e47777_app3.docx]

Appendix. Learning objectives for the *Teaching LGBTQ+ Health* course

*At the end of this continuing education activity, participants will be able to*:

1. Define common LGBTQ+ vocabulary for students
2. Discuss how social and behavioral determinants of queer health impact patient care
3. Choose appropriate instructional techniques to teach LGBTQ+ health to students
4. Explain potential challenges to healthcare access for LGBTQ+ patients
5. Role model techniques for eliciting a LGBTQ-inclusive sexual history
6. Design an inclusive clinical environment for both patients and learners
