# Supplementary material for: Teaching LGBTQ+ Health, a Web-Based Faculty Development Course: Program Evaluation Study Using the RE-AIM Framework
Source: JMIR Med Educ. 2023 Jul 21;9:e47777. doi: 10.2196/47777 (PMC10403800; doi:10.2196/47777)
Supplement: Multimedia Appendix 5 [file mededu_v9i1e47777_app5.docx]

Appendix. Curriculum Summary for the *Teaching LGBTQ+ Health* course

Section 1: Orientation

- Course Team
- Course Navigation
- Disclaimers and CME Credit Instructions

Section 2: Fundamentals of Teaching LGBTQ+ Health

- Introduction
- Pre-course Test
- LGBTQ+ Health Vocabulary
- Social and Behavioral Determinants of LGBTQ+ Health
- Teaching Strategies

Section 3: Teaching LGBTQ+ Health Cases

- Carla (case of a bisexual woman with a new cancer diagnosis)
- Carla Teaching Points
- Jesse (case of medical HIV prevention for a serodiscordant couple)
- Jesse Teaching Points
- Teddy (case of a non-binary patient seeking affirming gynecological care)
- Teddy Teaching Points

Section 4: Conclusions, Resources, and CME Credit Instructions

- Conclusion and Review
- Post-course Test
- References
- Teaching Resources
- Teaching LGBTQ+ Health CME Credit Instructions
