# Supplementary material for: Teaching LGBTQ+ Health, a Web-Based Faculty Development Course: Program Evaluation Study Using the RE-AIM Framework
Source: JMIR Med Educ. 2023 Jul 21;9:e47777. doi: 10.2196/47777 (PMC10403800; doi:10.2196/47777)
Supplement: Multimedia Appendix 7 [file mededu_v9i1e47777_app7.pdf]

CME Eval Questions:

1. I attest that I have completed the above activity.
2. On a scale from 1-10, with 1 being "Highly Unlikely" and 10 being "Highly Likely", how likely are you to recommend future Stanford CME activities to a friend or colleague?
3. Rate the following statements on a scale of 1-5
   a. Covered content useful to my practice.
   b. Contributed to my professional growth.
   c. Was relevant to my current scope of practice.
   d. Was engaging and interactive.
4. What changes do you intend to implement in your practice as a result of your participation in this CE/CME activity?
5. Was the information/material presented was balanced and free from commercial bias?
6. This CE/CME activity improved my knowledge, attitudes, and skills that support my inter- professional healthcare team and our delivery of patient care.
7. Do you have specific suggestions as to how this CE/CME activity might be improved?
8. How did you hear of this CE/CME activity?

Pre/Post Course Test Questions

Q1. Please assess your knowledge of LGBTQ+ health content. (This item is not scored)

- I have never heard of this content.
- I have heard of this content but don’t understand it.
- I have some understanding of this content.
- I understand this content quite well.
- I understand this content and could explain it to others.

Answer: (Item not scored, correct is always the response.)

Thank you for your response. Please advance to the next question.

Q2. Please assess your ability to teach LGBTQ+ health content. (This item is not scored)

- I cannot teach this content.
- I would like to teach this content, but I don’t know how to.

- I have a basic understanding of how to teach this content but do so rarely.
- I am confident enough to teach this content occasionally.
- I teach this content regularly.

Answer: (Item not scored, correct is always the response.)

Thank you for your response. Please advance to the next question.

Q3. Which of the following statements about LGBTQ+ health curricula at US medical schools is FALSE? (Choose all that apply.)

- Guidance from the Association of American Medical Colleges (AAMC) is inadequate.
- LGBTQ+ health content is included in 60% of US medical school curricula.
- Medical student demand for LGBTQ+ health curricula is high.
- Professional societies call for improved teaching of LGBTQ+ health.

Answer: "Guidance from the Association of American Medical Colleges (AAMC) is inadequate" and "LGBTQ+ health content is included in 60% of US medical school curricula" are not accurate statements. In 2014, the AAMC called for improvements to medical school climate and curriculum in a landmark publication that described core competencies for LGBTQ+ health education. However, LGBTQ+ health curricula are limited or non-existent at many US medical schools.

Location in Course: Introduction

This question tests knowledge of: the educational gap between demand for LGBTQ+ health content and expected faculty teaching competency.

Suggested Teaching Strategy: Curriculum Design. Seek opportunities to include LGBTQ+ health content in your clinical teaching and your school's curriculum.

Q4. Which of the following statements is FALSE? (Choose all that apply.)

- A gay male may be sexually or romantically attracted to a transmasculine person.
- "Lesbian" is a term that a patient may use to describe their gender identity.
- "Queer" is a term that may be used to describe gay men, lesbians, and/or transgender people.
- "Transfeminine" is a term that describes a person assigned the male sex at birth and who identifies as a woman or feminine non-binary person.

Answer: "Lesbian" is a term that a patient may use to describe their gender identity. "Lesbian" is a term that describes the sexual orientation of women who are mostly or exclusively attracted to other women. It does not explicitly describe an individual's gender. While gay men are generally attracted to other men, they may not be exclusively attracted to men. "Queer" is a reclaimed, positive, and inclusive term for all individuals who identify within the LGBTQ+ community, despite its historical use as a pejorative slur. "Transfeminine" and "transmasculine"– often abbreviated as "trans"– refer to a current gender expression that does not traditionally align with sex assignment at birth.

Location in Course: LGBTQ+ vocabulary/glossary

This question tests knowledge of: LGBTQ+ vocabulary.

Suggested Teaching Strategies: Proper use of these terms presents an opportunity to connect with LGBTQ+ patients by treating them with dignity and respect—and this is a great teaching point to emphasize to your students who are unfamiliar with this material.

Here are three strategies for teaching LGBTQ+ terminology: Item Writing. Use multiple-choice or true/false questions to confirm student knowledge of LGBTQ+ vocabulary. Curriculum Design. Use terms such as "Queer Health" and "LGBTQ+ Health" in your course syllabus. Clinical Teaching. Distinguish between gender, sexual orientation, and sex assignment at birth. Do not use the word "preferred" to describe a patient's gender, gender pronouns, or sexual orientation. Distinguish between gender, sexual orientation, and sex assignment at birth. Do not use the word "preferred" to describe a patient's gender, gender pronouns, or sexual orientation.

Q5. Which of the following social determinants of queer/trans health result in DECREASED utilization of primary care among LGBTQ+ patients? (Choose all that apply.)

- Familial Rejection
- Lack of Legal Protections
- Minority Stress
- Violence Against LGBTQ+ Individuals

Answer: All of these are social determinants. Social determinants of special importance to LGBTQ+ patients include Familial Rejection, Lack of Legal Protections, Minority Stress, and Violence Against LGBTQ+ Individuals. Each of these social determinants of queer health is well-documented in medical literature.

Location in Course: Social and Behavioral Determinants of LGBTQ+ Health

This question tests knowledge of: social determinants of LGBTQ+ health.

Suggested Teaching Strategy: Clinical Teaching. Review social and behavioral determinants of LGBTQ+ healthcare utilization when teaching students in your clinical practice.

Q6. Which of the following statements describing determinants of health care accessibility among LGBTQ+ individuals is TRUE? (Choose all that apply.)

- Intersecting minority identities have a synergistic effect on minority stress.
- Prevalence of substance use by sexual and gender minorities mirrors the general US population.
- Sexual and gender minority women access primary care similarly to other women.
- Unmarried domestic partners are considered legal health care proxies in all US states.

Answer: Intersecting minority identities have a synergistic effect on minority stress. Minority stress affects patient well-being and risks decreased engagement with health care providers. More than one minority status results in additive stress and increased health disparities.

Locations in Course: Social and Behavioral Determinants of LGBTQ+ Health, Case: Carla

This question tests knowledge of: social and behavioral determinants of LGBTQ+ healthcare utilization.

Suggested Teaching Strategy: Item Writing. Design multiple-choice, true/false, or essay questions that test student knowledge of various determinants of queer health. Use correct LGBTQ+ vocabulary in the stem of the question and the foils.

Q7. 'The 5 P's of Sexual Health' include all of the following EXCEPT:

- Partners
- Past sexually-transmitted infections
- Positions
- Practices
- Pregnancy Choices
- Protection from sexually-transmitted infections

Answer: Positions. The 5 P's of Sexual Health is a framework for obtaining a sexual history. One of the 5 questions prompts discussion of sexual practices, not positions. It is important to ask about sexual practices in order to specifically address the risks of sexually transmitted infections.

Locations in Course: Teaching Strategies, Case: Jesse

This question tests knowledge of: important elements of a sexual history.

Suggested Teaching Strategy: Role Modeling. Demonstrate using the '5 P's of Sexual Health' when teaching students how to obtain a sexual history during a patient interview. Your students may be uncomfortable with this technique initially. Role model the technique for them, observe their performance and provide feedback during your debriefing.

Q8.You are caring for a serodiscordant gay couple. The patient who is HIV+ positive is compliant with HAART (Highly Active Anti Retroviral Therapy) and has an undetectable HIV viral load. Your trainee uses the '5 P's of Sexual Health' technique of obtaining a sexual history, learns that the couple does not use condoms during penetrative anal sex, and asks you about HIV prevention counseling. What is the risk of HIV transmission for this couple?

- 0%
- 15%
- 50%
- 85%
- 100%

Answer: 0% In accordance with the PARTNER2 study, when the HIV+ partner has an undetectable viral load, the risk of HIV transmission to the HIV- partner is effectively 'no risk'.

Locations in Course: Teaching Strategies, Case: Jesse

This question tests knowledge of: HIV transmission risk for serodiscordant sexual partners.

Suggested Teaching Strategy: Clinical Teaching. Teach your students that 'Undetectable = Untransmittable.'

Q9. Which of the following statements about transgender patients is FALSE? (Choose all that apply.)

- All transgender individuals seek gender-affirming medications and surgical interventions.
- Transgender individuals seek primary care as frequently as other individuals.
- Transmasculine patients seek cervical cancer screening as frequently as other individuals.

Answer: All 3 statements are false. Transgender patients may never seek gender-affirming medications or surgical procedures. Do not assume that all transgender patients want to pursue (or already had) a gender-affirming surgical procedure. Transgender patients access primary care services less frequently than the general population. Transmasculine patients both seek and are offered cervical cancer screening less frequently than the general population.

Locations in Course: Case: Teddy

This question tests knowledge of: common health-seeking behaviors of transgender patients.

Suggested Teaching Strategy: Clinical Teaching. Teach your students about healthcare utilization patterns and disease prevention counseling for LGBTQ+ individuals.

Q10. Which of the following changes would make for a more affirming clinical practice environment for LGBTQ+ patients, staff, and students? (Check all that apply.)

- Designating ‘All Gender Restrooms’ with appropriate signage
- Posting the LGBTQ+ Healthcare Bill of Rights in your waiting room
- Posting several easily visible ‘rainbow flags’ throughout the clinic
- Renaming your OBGYN practice a ‘Women’s Clinic’

Answer: Design elements of a medical clinic that are welcoming and inclusive of queer patients include ‘all gender’ restroom signs, care affirming statements such as the LGBTQ+ Healthcare Bill of Rights, rainbow flags, and gender-inclusive medical forms. Some transgender patients avoid practices that are labeled ‘Women’s Clinic.’

Locations in Course: Case: Teddy

This question tests knowledge of: inclusive clinical environments for patients and students.

Suggested Teaching Strategy: Clinical Teaching. Engage staff and students when redesigning your clinical workplace. Seek feedback and suggestions from LGBTQ+ patients in your practice.

Pre/Post Test Assessment Report - per entry/user

1. Date of test completion
2. % correct
3. % incorrect

4. Total points
5. Results %

Course Report Reporting Categories

1. Individual User Progress %
2. Individual User Completed Steps
3. Individual User Completed on (date)
4. Total Users Not Started
5. Total Users In-Progress
6. Total Users Completed
