# Supplementary material for: Teaching LGBTQ+ Health, a Web-Based Faculty Development Course: Program Evaluation Study Using the RE-AIM Framework
Source: JMIR Med Educ. 2023 Jul 21;9:e47777. doi: 10.2196/47777 (PMC10403800; doi:10.2196/47777)
Supplement: Multimedia Appendix 10 [file mededu_v9i1e47777_app10.pdf]

Stanford MEDICINE

NEW ONLINE COURSE:

TEACHING LGBTQ+ HEALTH

A Faculty Development Course for Health Professions Educators

PALO ALTO, MARCH 22, 2021 - Today's medical curriculum is lacking in substantive LGBTQ+ patient representation. Medical students have expressed a need for a more well-rounded approach to training for LGBTQ+ patient care and medical educators have expressed a lack of confidence to teach these topics. The Stanford Medicine **Teaching LGBTQ+ Health Topics: A Faculty Development Course for Health Professions Educators** aims to give health educators the instruction they need to bridge the gap between what is needed and what is presently offered . The materials in this course are evidence-based and practice-driven. They offer best practices and tools for easy implementation into existing curriculum. The content has been vetted by medical educators and LGBTQ+ health topics subject matter experts. The authors and actors who have lent their clinical experiences and voices to build these materials have come from the LGBTQ+ community and are direct reflections of the needs for improved LGBTQ+ patient care. The course consists of 7 interactive modules including a **Glossary** of LGBTQ+ health-relevant terms, an overview of the **Social and Behavioral Determinents** our LBGTQ+ patients face, best practices for **Teaching Strategies**, and **3 Cases** with teaching points built in to help educators incorporate similar topics in their own curricula.

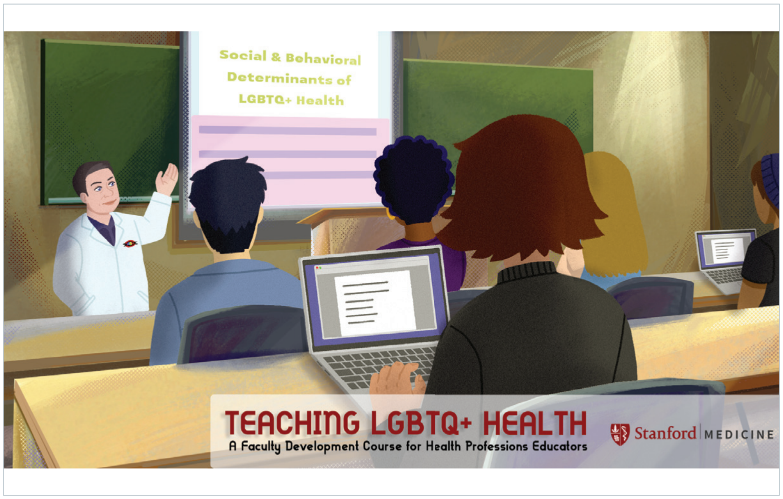

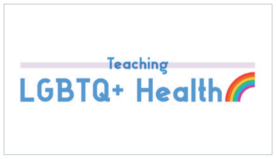

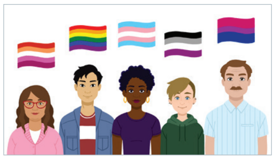

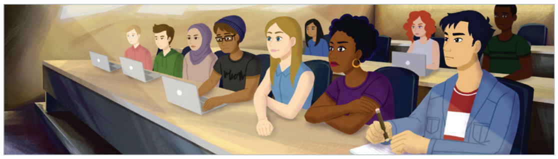

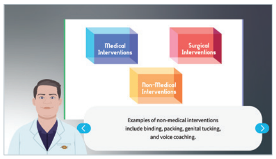

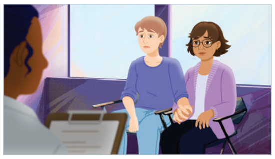

LINKS

TRAILER:
STANFORD MEDEDUCATION:
CLOUDCME:
